# Effect of instant jasmine rice coating combining *Spirulina* with edible polymers on physicochemical properties, textural properties and sensory acceptance

**DOI:** 10.1038/s41598-022-11759-8

**Published:** 2022-05-11

**Authors:** Niramon Utama-ang, Ittikorn Kuatrakul, Ponjan Walter, Panida Rattanapitigorn, Arthitaya Kawee-ai

**Affiliations:** 1 Division of Product Development Technology, Faculty of Agro-Industry, Chiang Mai University, Chiang Mai, 50100 Thailand; 2 Cluster of High Value Product from Thai Rice and Plants for Health, Chiang Mai University, Chiang Mai, 50100 Thailand; 3 Cluster of Innovative Food and Agro-Industry, Chiang Mai University, Chiang Mai, 50100 Thailand; 4 Division of Food Science and Technology, Faculty of Agro-Industry, Chiang Mai University, Chiang Mai, 50100 Thailand

**Keywords:** Biochemistry, Biotechnology

## Abstract

Coating is an effective and economic strategy to increase the functional property of food products. This study investigated the technical feasibility of adding *Spirulina platensis* to edible polymers, namely carboxymethyl cellulose (CMC) and maltodextrin (MD), in the coating of instant jasmine rice, using a central composite design (CCD). A total of 10 edible coating formulations comprising CMC (10–30% w/v) and MD (1–5% w/v) were evaluated to optimize the most suitable combination of physicochemical properties, textural attributes, and sensory acceptance. The resulting rice fortified with *S. platensis* and hydrocolloids showed improved textural and functional properties favourable for consumer acceptance. Among these, the optimum (20.0% MD, 1.0% CMC, and 2.0% *S. platensis* powder) increased the physicochemical properties and decreased textural properties compared with those of uncoated rice. This condition showed phycocyanin content of 1.4 mg/g, chlorophyll a of 181.5 µg/g, total phenolic compound (TPC) of 137.3 µg gallic acid equivalent (GAE)/g, and ferric reducing antioxidant power (FRAP) of 3.8 mg ferrous (Fe^2+^)/g with overall acceptability of 7.1 (like moderately). It can be stated that masking the colour and flavour of *Spirulina* with an edible coating could be a healthy alternative to commercial rice and used to fortify cereal products with algae.

## Introduction

In recent years, coating technology has received much attention from researchers. Edible coating is incorporated directly into food products in a liquid form, followed by drying^1^. The coating materials are food-grade suspensions, the major components of which are hydrocolloids (polysaccharides and proteins) and lipids. Maltodextrin (MD) is a hydrolysed starch commonly used as a wall material in food ingredients, which offers a relatively low cost, neutral aroma and taste, and oxidation protection^2^. However, MD has a low emulsifying capacity, thus its use in combination with other active polymers is considerable. Carboxymethyl cellulose (CMC) is an anionic derivative of cellulose and has good water solubility, film-forming capacity, adhesion, biocompatibility, and biodegradability^3^. CMC can lower flavour release and improve surface properties^4^.

Rice (*Oryza sativa* L.) is one of the most consumed cereal plants, which half of the global population consumes as a staple food. Hom Mali 105 or Thai jasmine rice is the most popular variety in Thailand due to its aroma and tender texture and is often used for producing instant rice^5^. The blue-green microalga *Spirulina platensis* has been consumed as a food and a nutritional supplement due to being rich in micro-and macro-nutrients—for example, vitamins, amino acids, polypeptides, phytohormones, and polyunsaturated fatty acids^6^. *Spirulina* is a generally recognized as safe (GRAS) ingredient with no toxicological effect and is approved by the Food and Drug Administration (FDA)^7^. In addition, this microalga contains phycocyanin which can protect against oxidative stress-induced diseases^8^.

Rice analogue, also referred to as artificial rice, rice substitutes, alternative rice, is suitable for some people with special dietary needs and provide health benefits by delivering various bioactive compounds^9^. Fortified nutrients, which lack in real rice, can be added during the manufacturing process and resulting in nutritional benefits. Currently, there is more demand for instant rice products gain from consumers due to their convenience. Numerous studies have investigated the improvement of instant rice product quality by developing additives and cooking, dehydration, and drying processes^10–12^. Furthermore, fortification with *S. platensis* can increase the functional properties of yogurt^13^ and pasta^14^. However, information about the application of coating technology to instant rice has been limited.

As mentioned above, this study investigated the effects of a coating made from *S. platensis* in combination with hydrocolloids (MD and CMC) on the physicochemical and textural properties, and sensory evaluation of instant rice, while response surface methodology was applied to optimize the improvement of instant rice quality.

## Results and discussion

### Fitting the model

The effect of independent variables on the physicochemical properties, texture properties, and sensory analysis is presented in Table 1a–c, respectively. The response surface models were utilized to predict the linear, quadratic, and interaction effects of polymers on all 23 attributes in combination with *S. platensis*; however, only 10 attributes were the significant variables (Table 2). Regression equations of the significant variables, obtained by RSM, are shown in terms of actual factors. According to ANOVA, MD had a significant effect (*p* < 0.05) for the linear parameters on coating efficiency (CE), texture properties (hardness and gumminess) and sensory attributes (appearance and colour), while CMC had a significant effect on the physicochemical properties (L* and b*) and colour attributes of sensory evaluation.

**Table 1:** Operation parameters and physicochemical properties, textural properties, and sensory acceptance responses of coated instant rice by CCD.

Codes		Variables		a. Physicochemical properties responses								
A	B	MD (%)	CMC (%)	L*	a*	b*	CE (%)	Phycocyanin (mg/g)	Chlorophyll a (µg/g)	TPC (µg GAE/g)	FRAP (mg Fe^2+^/g)	
− 1	− 1	10	1	26.0 ± 2.0	− 1.9 ± 0.6	7.9 ± 0.5	63.3 ± 0.2	1.9 ± 0.2	202.6 ± 1.9	95.3 ± 4.0	3.3 ± 0.1	
− 1	+ 1	10	5	31.7 ± 2.4	− 2.4 ± 0.7	10.4 ± 0.6	65.3 ± 0.5	2.1 ± 0.2	213.8 ± 0.4	98.1 ± 6.3	3.0 ± 0.0	
+ 1	− 1	30	1	24.7 ± 5.5	− 3.8 ± 0.9	8.4 ± 0.5	73.2 ± 1.2	2.2 ± 0.1	218.1 ± 0.7	115.4 ± 5.5	3.4 ± 0.0	
+ 1	+ 1	30	5	33.1 ± 1.2	− 3.3 ± 0.3	11.3 ± 0.4	72.6 ± 0.1	2.4 ± 0.1	212.1 ± 1.7	102.9 ± 5.2	3.7 ± 0.1	
+ α	0	34.1	3	32.3 ± 1.4	− 2.7 ± 0.3	10.4 ± 0.6	73.5 ± 0.9	1.8 ± 0.2	210.7 ± 0.0	106.5 ± 4.0	3.6 ± 0.1	
− α	0	5.8	3	28.1 ± 1.7	− 3.1 ± 0.5	8.4 ± 0.4	63.3 ± 0.8	1.9 ± 0.1	203.4 ± 3.4	101.9 ± 8.1	3.3 ± 0.1	
0	+ α	20	5.8	33.4 ± 1.2	− 2.3 ± 0.3	10.7 ± 0.5	74.0 ± 0.4	2.2 ± 0.1	176.1 ± 2.4	109.8 ± 0.0	4.1 ± 0.1	
0	− α	20	0.2	26.0 ± 1.3	− 1.3 ± 0.5	7.7 ± 0.7	75.0 ± 1.1	3.2 ± 0.2	227.0 ± 0.6	129.7 ± 6.2	4.0 ± 0.1	
0	0	20	3	28.6 ± 0.9	− 3.0 ± 0.8	9.3 ± 0.6	72.6 ± 0.1	2.9 ± 0.1	212.1 ± 1.2	132.4 ± 7.4	4.4 ± 0.1	
0	0	20	3	29.0 ± 2.2	− 3.4 ± 0.9	9.5 ± 0.4	73.2 ± 0.4	2.8 ± 0.1	208.9 ± 0.5	139.2 ± 13.7	4.4 ± 0.0	

Codes		Variables		b. Texture properties responses								
A	B	MD (%)	CMC (%)	Hardness (N)	Adhesiveness (N)	Cohesiveness	Springiness (mm)	Gumminess (mJ)	Chewiness (mJ)			
− 1	− 1	10	1	42.4 ± 5.2	− 2.0 ± 0.6	0.51 ± 0.08	0.18 ± 0.03	21.8 ± 3.5	3.9 ± 0.7			
− 1	+ 1	10	5	36.0 ± 9.5	− 2.2 ± 0.6	0.44 ± 0.03	0.16 ± 0.02	15.7 ± 1.5	2.4 ± 0.3			
+ 1	− 1	30	1	38.0 ± 7.2	− 1.9 ± 0.5	0.47 ± 0.02	0.16 ± 0.04	17.6 ± 2.9	2.9 ± 0.6			
+ 1	+ 1	30	5	28.5 ± 3.8	− 1.7 ± 0.5	0.48 ± 0.02	0.15 ± 0.02	13.5 ± 1.8	2.0 ± 0.4			
+ α	0	34.1	3	34.3 ± 10.6	− 1.6 ± 0.5	0.47 ± 0.03	0.15 ± 0.02	15.7 ± 4.7	2.4 ± 0.6			
− α	0	5.8	3	41.4 ± 3.8	− 2.0 ± 0.2	0.49 ± 0.03	0.17 ± 0.03	19.9 ± 5.8	3.1 ± 0.9			
0	+ α	20	5.8	35.2 ± 5.8	− 1.5 ± 0.6	0.50 ± 0.04	0.16 ± 0.03	17.8 ± 5.1	2.8 ± 0.8			
0	− α	20	0.2	33.9 ± 5.4	− 1.0 ± 0.4	0.52 ± 0.05	0.13 ± 0.03	17.3 ± 4.7	2.4 ± 0.8			
0	0	20	3	35.6 ± 7.4	− 1.6 ± 0.2	0.45 ± 0.04	0.18 ± 0.03	16.0 ± 1.5	2.9 ± 0.4			
0	0	20	3	36.1 ± 5.7	− 1.6 ± 0.2	0.47 ± 0.04	0.16 ± 0.02	16.3 ± 2.9	2.6 ± 0.7			

Codes		Variables		c. Sensory acceptance responses								
A	B	MD (%)	CMC (%)	Appearance	Colour	Seaweed aroma	Overall taste	Seaweed flavour	Rice flavour	Softness	Adhesiveness	Overall acceptability
− 1	− 1	10	1	5.9 ± 1.2	5.2 ± 1.3	5.5 ± 0.9	5.8 ± 1.0	5.4 ± 0.9	6.4 ± 0.8	6.7 ± 0.8	6.3 ± 1.0	6.2 ± 1.1
− 1	+ 1	10	5	6.0 ± 1.0	5.5 ± 1.3	5.6 ± 1.0	6.1 ± 1.0	5.6 ± 1.1	6.3 ± 1.1	6.4 ± 1.3	6.0 ± 1.2	6.0 ± 1.1
+ 1	− 1	30	1	5.7 ± 1.3	5.6 ± 1.4	5.3 ± 1.3	5.9 ± 1.3	5.4 ± 1.3	6.2 ± 1.1	6.4 ± 1.1	6.2 ± 1.4	6.1 ± 1.3
+ 1	+ 1	30	5	5.9 ± 1.1	6.2 ± 1.1	5.6 ± 1.1	5.7 ± 1.1	5.4 ± 1.2	6.2 ± 0.9	6.1 ± 0.9	6.2 ± 0.9	5.7 ± 1.3
+ α	0	34.1	3	5.8 ± 1.1	6.0 ± 1.0	5.8 ± 1.1	6.1 ± 0.9	5.9 ± 1.0	6.4 ± 0.9	6.4 ± 1.0	6.1 ± 1.4	6.3 ± 1.1
− α	0	5.8	3	6.3 ± 1.1	5.6 ± 1.2	5.7 ± 1.2	5.8 ± 1.0	5.7 ± 1.1	6.2 ± 0.8	6.2 ± 0.9	6.2 ± 0.9	6.0 ± 1.0
0	+ α	20	5.8	5.8 ± 1.2	5.7 ± 1.2	5.6 ± 0.9	5.8 ± 0.9	5.8 ± 1.3	6.3 ± 0.8	6.8 ± 0.7	6.6 ± 0.9	6.3 ± 1.0
0	− α	20	0.2	5.7 ± 1.2	5.0 ± 1.4	5.5 ± 1.3	5.9 ± 1.2	5.6 ± 1.2	6.1 ± 0.8	6.8 ± 1.0	6.5 ± 1.0	6.3 ± 1.2
0	0	20	3	6.2 ± 1.0	6.1 ± 1.1	5.7 ± 1.0	6.3 ± 0.9	5.9 ± 0.8	6.2 ± 0.9	6.5 ± 1.0	6.3 ± 1.1	6.0 ± 1.1
0	0	20	3	6.1 ± 1.0	6.0 ± 1.1	5.7 ± 0.9	6.1 ± 1.1	5.6 ± 0.9	6.3 ± 0.9	6.7 ± 0.9	6.3 ± 1.1	6.0 ± 1.1

**Table 2:** Regression coefficients of the models for available attributes.

Response	Model	*p*-value	*Adj-R*^2^	Lack of fit
**Physicochemical attributes**				
L*	+ 23.2 + 0.07*MD + 1.5*CMC	0.0005	0.8498	0.1901
b*	+ 6.6 + 0.05*MD + 0.6*CMC	< 0.0001	0.9495	0.3620
CE (%)	+ 51.5 + 1.7* MD + 0.5* CMC-0.0.3* MD^2^ + 0.02*CMC^2^ -0.03 MD * CMC	0.0352	0.7882	0.1154
Phycocyanin (mg/g)	+ 0.9 + 0.2*MD-0.07*CMC-5.0 × 10^–3^*MD^2^	0.0411	0.5855	0.1990
TPC (µg GAE/g)	+ 39.7 + 7.6*MD + 13.0*CMC-0.2*MD^2^-2.6*CMC^2^	0.0284	0.7254	0.3919
FRAP (mg Fe^2+^/g)	+ 1.1 + 0.2*MD + 0.5*CMC-0.6*10^–3^*MD^2^-0.1*CMC^2^	0.0442	0.6686	0.1647
**Texture attributes**				
Hardness (N)	+ 44.2–0.3*MD-0.9*CMC	0.0362	0.5018	0.0887
Gumminess (mJ)	+ 22.02–0.1*MD-0.6*CMC	0.0358	0.5035	0.1008
**Sensory attributes**				
Appearance	+ 5.8 + 6.7 × 10^–3^ * MD + 0.3* CMC-6.1 × 10^–3^ * MD^2^-0.05 * CMC^2^ + 2.0 × 10^–3^ * MD * CMC	0.0259	0.8195	0.4974
Colour	+ 4.4 + 0.05* MD + 0.5* CMC-1.0 × 10^–3^* MD^2^-0.08 * CMC^2^ + 3.5 × 10^–3^ * MD * CMC	0.0094	0.8933	0.3518

**Figure 1:**
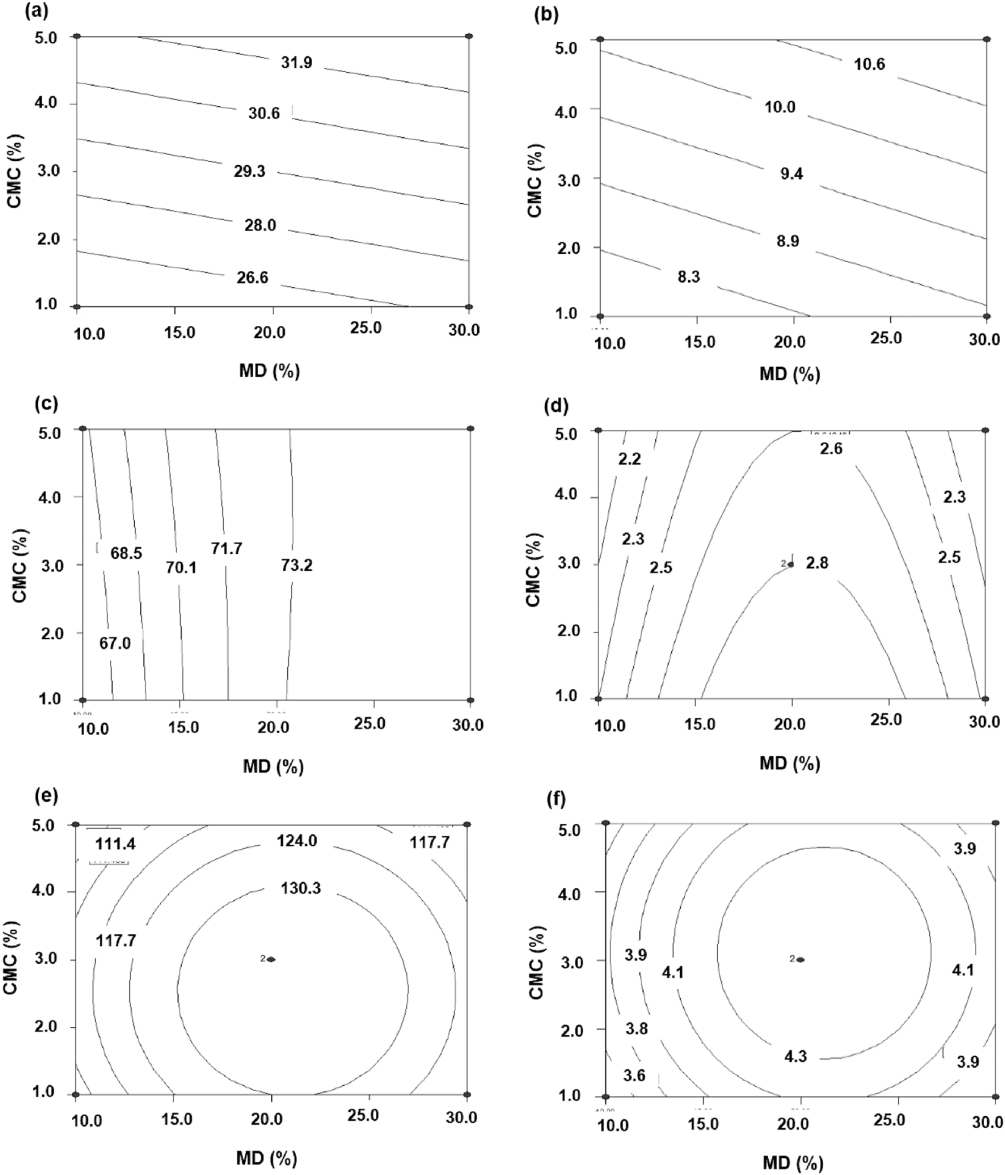
Contour plots of the effect of the interaction between MD and CMC on physiochemical attributes; L* (**a**), b* (**b**), CE (**c**), phycocyanin (**d**), TPC (**e**), and FRAP (**f**).

Two-dimensional plots were created in order to illustrate the response surfaces for the intended models. From Table 2 and Fig. 1, it can be seen that CMC had a significant positive impact on L* and b* values (Fig. 1a,b), as CMC was responsible for forming intermolecular bonds with *S. platensis*. A rise of CMC content significantly increased the L* and b* values. The optimal concentrations of MD and CMC for coating instant rice with a high CE, total phenolic compounds (TPC), FRAP, and phycocyanin were in the range of 15–25% and 1–3%, respectively (Fig. 1c–f). The high CE might be due to the interaction of MD, CMC, and protein–polysaccharide^15^.

**Figure 2:**
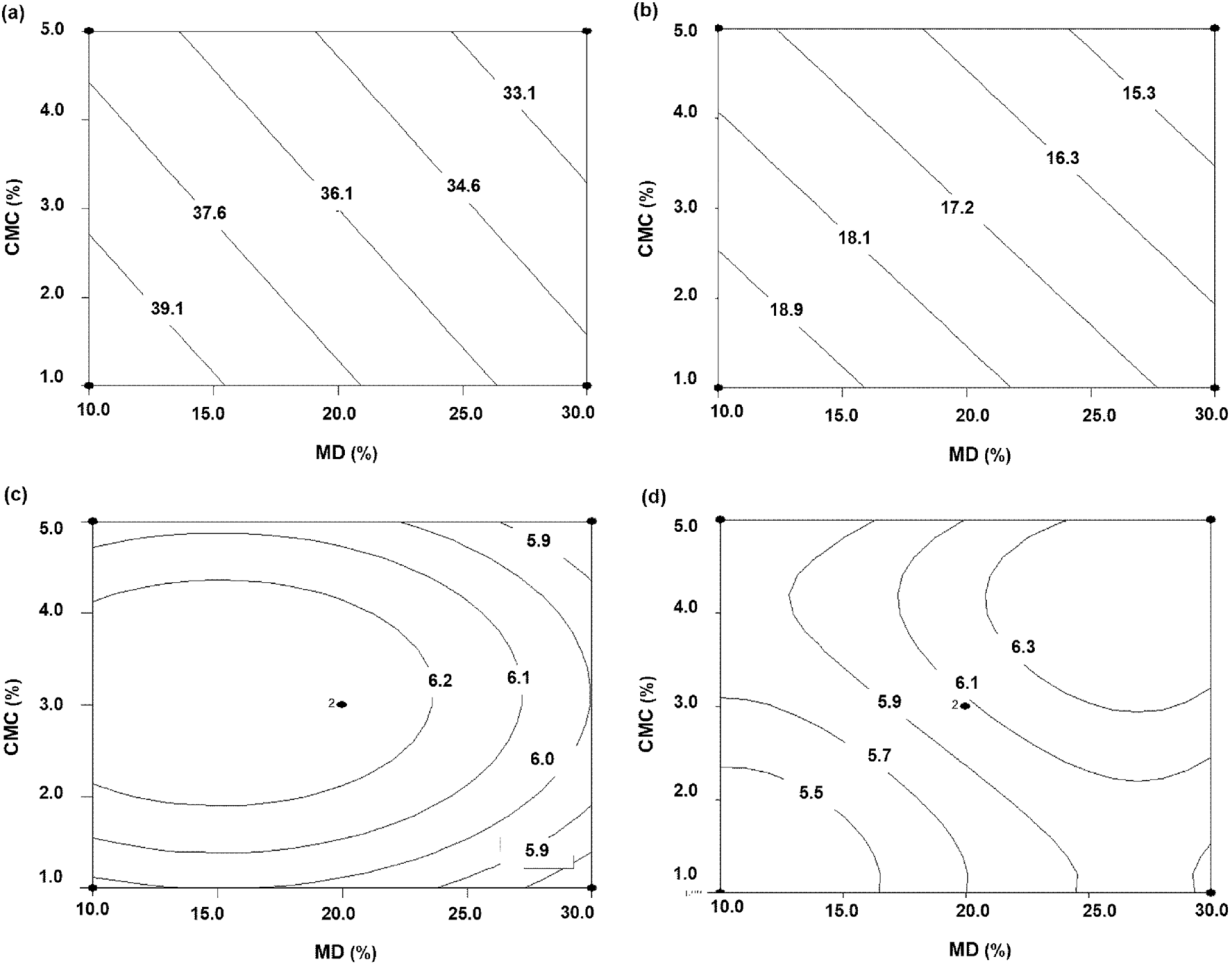
Contour plots of the effect of the interaction between MD and CMC on texture and sensory attributes; hardness (**a**), gumminess (**b**), appearance (**c**), and colour (**d**).

Meanwhile, an increase of MD content significantly decreased (*p* < 0.05) the hardness and gumminess of the instant rice (Fig. 2a,b), which may be due to the hydrocolloids increasing the hydrophilic properties of the instant rice surface. Unlike what was observed for the physicochemical and texture properties, the inclusion of > 20% MD (w/v) and > 3% CMC (w/v) significantly increased the product’s hedonic score for appearance and colour (Fig. 2c,d).

**Figure 3:**
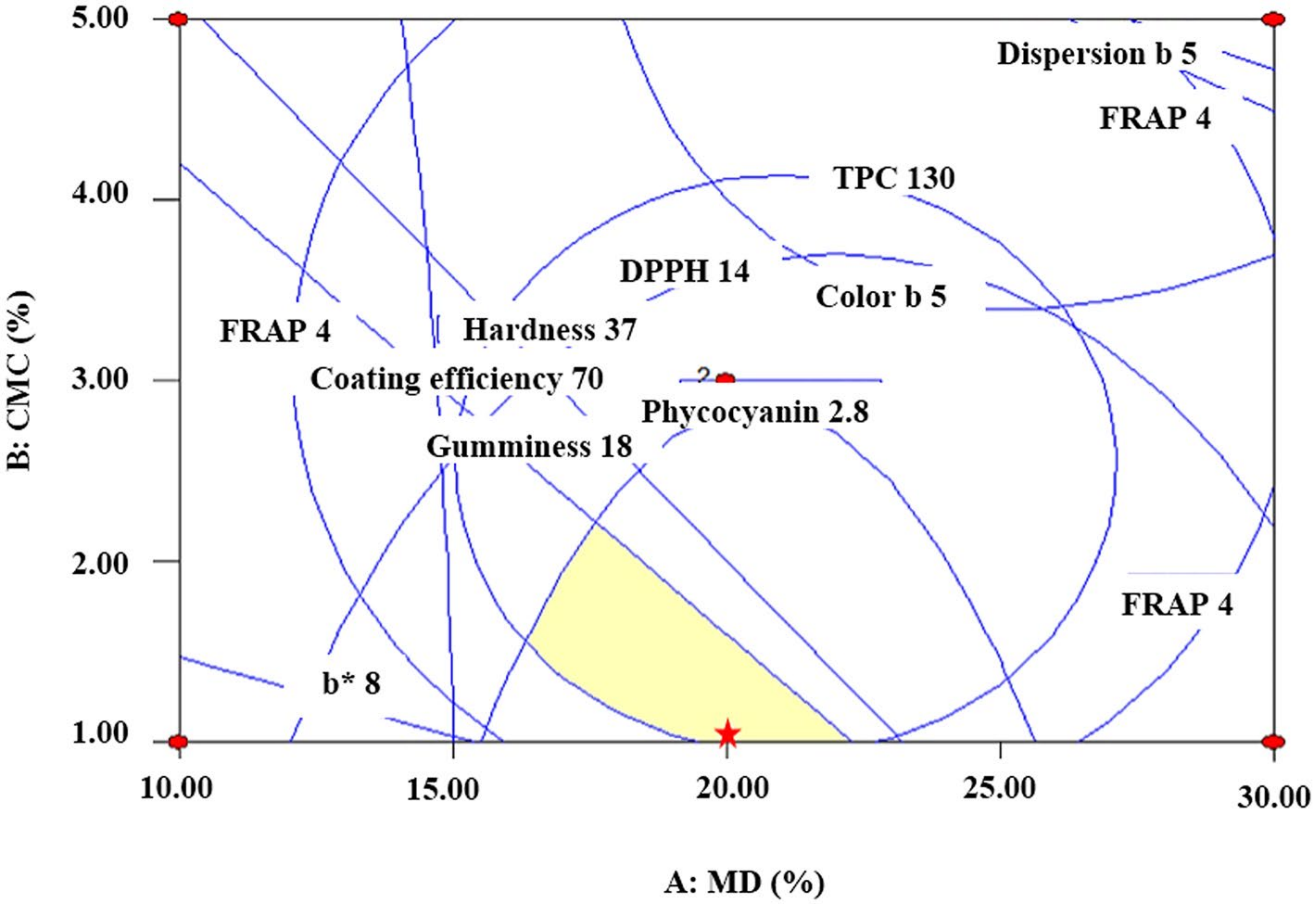
Overlay plot for the optimal concentration for edible coating instant rice with *S. platensis*.

The optimum concentrations of MD and CMC in combination with *S. platensis* to reach the highest L*, b*, TPC, FRAP, phycocyanin, appearance, and colour scores; and those for other variables like CE, hardness, and gumminess (in a range) were determined by solving the equation in Table 2, using Design-Expert software. The MD and CMC values were estimated as 20.0% and 1.0%, respectively, with desirability of 0.762 (Fig. 3).

### Effect of hydrocolloids on instant rice coating

#### Change in proximate composition

The proximate composition of the investigated materials was evaluated on a dry weight (DW) basis and is presented in Table 3. The rice was rich in carbohydrate (82.0 ± 0.4%), followed by protein (9.3 ± 0.55%), whereas *S. platensis* was rich in protein (69.9 ± 2.0%). The ash content indicates the total mineral content present in the sample, which means that *S. platensis* (5.3 ± 0.5%) had a higher mineral content than that of jasmine rice (1.5 ± 0.1%). The minerals present in *S. platensis* are potassium, phosphorus, calcium, magnesium, iron, manganese, zinc, boron, and copper^16^, while iron, phosphorus, and calcium are represented in jasmine rice^17^. Phycocyanin and chlorophyll a are the main phytopigments in *S. platensis*^16^. In this study, the phycocyanin and chlorophyll a content observed in *S. platensis* was 22.4 ± 0.2% and 7.8 ± 0.5%, respectively.

**Table 3:** Proximate composition of rice, *S. platensis*, and coated instant rice.

Composition (%)	Samples*		
	Rice (Control)	*S. platensis*	Coated instant rice
**Proximate**			
Moisture	4.6 ± 0.1^b^	4.5 ± 0.9^b^	7.7 ± 0.2^a^
Protein	9.3 ± 0.5^c^	69.9 ± 2.0^a^	15.3 ± 0.8^b^
Carbohydrate	82.0 ± 0.4^a^	17.9 ± 0.9^c^	72.5 ± 0.7^b^
Fat	1.4 ± 0.0^b^	0.6 ± 0.1^c^	1.8 ± 0.1^a^
Fibre	1.2 ± 0.0^a^	2.2 ± 0.5^a^	1.4 ± 0.1^b^
Ash	1.5 ± 0.1^b^	5.3 ± 0.5^a^	1.3 ± 0.1^b^
**Active compound**			
Phycocyanin	nd**	22.4 ± 0.2^a^	15.7 ± 0.4^b^
Chlorophyll a	nd	7.8 ± 0.5^a^	1.8 ± 0.1^b^

*a–c represented the significant difference in the rows at *p* < 0.05.

**nd means not detected.

The coated instant rice had a much higher protein content (15.3 ± 0.8%), up to 64.5% higher than that of the control. The increase of protein content might be due to a high concentration of macromolecule-like protein in *S. platensis*^6^. Due to the hydrophilic nature of the protein, CMC, and MD, a high moisture content of the coated instant rice was obtained. The moisture content of the coated instant rice increased up to 67.4% when CMC and MD were applied compared to the control. Coating materials serve as physical barriers to prevent the evaporation of moisture. CMC contains carboxymethyl groups (–CH_2_–COONa) with excellent water absorption capacity^3^ that may be bonded to the hydroxyl groups (–OH) of phycocyanin and other active compounds of *S. platensis*. The fat and fibre content of the rice increased slightly on enrichment of the coating materials with *S. platensis*. The reduction of ash content might be due to the loss of phycocyanin and chlorophyll a, and other minerals.

### Changes in physicochemical properties

#### Colour

Table 4 shows the physicochemical and texture properties, together with the results from sensory analysis of the uncoated instant rice and that coated with hydrocolloids plus *S. platensis* (1%, 2%, and 3% (w/v)). The appearance of uncoated instant rice and that coated with MD, CMC, and *S. platensis* at 1%, 2%, and 3% (w/v) is presented in Table 4. An increase of *S. platensis* level resulted in increased green colour, which is proved by the colour analysis. A change in food colour might decrease product quality, marketing value, and consumer acceptability. A higher L* value represents a brighter product, while positive and negative a* and b* values represent red to green and yellow to blue colours, respectively. In this study, the L* of coated instant rice tended to decrease with increasing *S. platensis* levels, indicating that the coated rice was darkened by *S. platensis*, which also resulted in an increase of the b* values. The lower a* value of coated instant rice means the colour of the product had become greener. Enrichment with *S. platensis* increased the yellow (b* value) and green (a* value) colour of the instant rice; this result is in agreement with those for the fortification with *S. platensis* of yogurt^13^, pasta^14^, and soy yogurt^18^.

**Table 4:** Comparative of physicochemical properties, texture properties and sensory acceptance of uncoated and coated instant rice with different concentration of *Spirulina.*

Properties	Uncoated instant rice	Coated instant rice fortified with S. platensis**				
		1% (w/v)	2% (w/v)	3% (w/v)	4% (w/v)	5% (w/v)
Appearance						
**Physicochemical**						
L*	57.0 ± 1.8	54.4 ± 0.9^a^	37.7 ± 0.6^b^	36.8 ± 2.2^b^	35.0 ± 0.5^c^	34.7 ± 1.2^c^
a*	− 1.0 ± 0.0	− 2.2 ± 0.1^a^	− 3.5 ± 0.3^bc^	− 3.3 ± 0.2^b^	− 3.6 ± 0.1^c^	− 4.3 ± 0.3^d^
b*	7.8 ± 1.3	16.8 ± 1.6^a^	10.6 ± 0.2^b^	8.0 ± 0.5^c^	10.2 ± 0.5^b^	4.3 ± 0.8^d^
Phycocyanin (mg/g)	nd***	0.8 ± 0.0^d^	1.4 ± 0.07^c^	2.7 ± 0.01^a,b^	2.6 ± 0.0^b^	3.0 ± 0.2^a^
Chlorophyll a (µg/g)	nd	126.1 ± 2.1^d^	181.5 ± 3.6^c^	261.5 ± 7.0^b^	358.1 ± 16.6^a^	260.3 ± 5.6^a^
TPC (µg GAE/g)	nd	102.1 ± 5.3^e^	137.3 ± 3.9^d^	208.5 ± 8.0^c^	285.9 ± 7.5^b^	315.9 ± 11.9^a^
FRAP (mg Fe^2+^/g)	nd	1.6 ± 0.1^c^	3.8 ± 0.7^b^	4.2 ± 0.0^b^	6.7 ± 0.0^a^	6.8 ± 0.2^a^
**Texture**						
Hardness^ns^* (N)	112.8 ± 14.4	22.0 ± 2.5	28.1 ± 6.9	31.0 ± 9.5	27.6 ± 11.0	34.7 ± 15.5
Adhesiveness^ns^ (N)	− 4.1 ± 1.1	− 0.4 ± 0.1	− 0.9 ± 0.4	− 1.0 ± 0.5	− 0.6 ± 0.4	− 0.9 ± 0.4
Cohesiveness^ns^	0.5 ± 0.0	0.5 ± 0.1	0.6 ± 0.1	0.5 ± 0.0	0.5 ± 0.1	0.5 ± 0.0
Springiness^ns^ (mm)	0.3 ± 0.0	0.2 ± 0.0	0.2 ± 0.0	0.2 ± 0.0	0.2 ± 0.0	0.2 ± 0.0
Gumminess^ns^ (mJ)	59.7 ± 7.4	11.3 ± 1.0	15.3 ± 4.7	15.4 ± 4.6	13.4 ± 5.0	15.8 ± 6.2
Chewiness^ns^ (mJ)	19.1 ± 3.6	1.7 ± 0.2	2.4 ± 0.9	3.0 ± 1.2	2.2 ± 1.1	2.6 ± 1.3
**Sensory**						
Appearance	7.0 ± 1.0	5.8 ± 1.2^c^	7.2 ± 1.1^a^	6.2 ± 1.2^b,c^	6.4 ± 1.0^b^	5.1 ± 0.9^d^
Colour	7.0 ± 1.0	5.5 ± 1.2^c^	6.9 ± 1.3^a^	6.5 ± 1.2^a,b^	6.3 ± 1.3^b^	4.8 ± 1.0^d^
Seaweed aroma	–	5.1 ± 1.1^c^	6.7 ± 1.4^a^	6.0 ± 1.2^b^	6.2 ± 1.1^a,b^	5.5 ± 1.1^c^
Overall taste	6.5 ± 1.1	6.1 ± 1.1^b,c^	6.7 ± 1.0^a^	6.6 ± 1.0^a^	6.4 ± 0.8^a,b^	5.7 ± 0.9^c^
Seaweed flavour	–	5.8 ± 1.1^b,c^	6.5 ± 1.0^a^	6.0 ± 1.1^b,c^	6.1 ± 0.9^a,b^	5.6 ± 0.8^c^
Rice flavour	6.7 ± 1.1	6.5 ± 1.1^b^	7.0 ± 1.1^a^	6.4 ± 0.8^b^	6.1 ± 0.7^bc^	5.9 ± 0.6^c^
Softness	6.4 ± 1.5	6.6 ± 1.1^b^	7.3 ± 1.0^a^	6.6 ± 0.8^b^	6.4 ± 0.6^bc^	6.1 ± 0.9^d^
Adhesiveness^ns^	6.5 ± 1.2	6.7 ± 0.9	6.8 ± 1.1	6.5 ± 0.8	6.5 ± 0.7	6.3 ± 0.9
Overall acceptability	6.6 ± 1.0	6.1 ± 1.2^c,d^	7.1 ± 1.1^a^	6.6 ± 1.1^b^	6.4 ± 1.2^b,c^	5.7 ± 1.1^d^

*ns means non-significant at *p* < 0.05.

**a–c represented the significant difference in the rows of coated instant rice at *p* < 0.05.

***nd means not detected.

### Phycocyanin, chlorophyll a, TPC, and antioxidant activity

The addition of *S. platensis* led to significant increases in the coated instant rice’s content of bioactive compounds like phycocyanin, chlorophyll a, and TPC (*p* < 0.05), as presented in Table 4. The bioactive compounds increased with an increase of *S. platensis* concentration. Phycocyanin, a blue pigment, is a phycobiliprotein or protein–pigment complex, which exerts anti-tumour, anti-inflammatory, anticancer, and antioxidant effects^19^. Chlorophyll a, a green pigment, plays a role as a natural toxin cleaner, antioxidant activity, and anti-ageing agent^9^. Therefore, the coating of *S. platensis* on the instant rice surface can improve health due to diverse functional compounds presented in *Spirulina*. The increase of TPC was due to the increase of phycocyanin and chlorophyll a contents, which resulted in increased antioxidant activity. The antioxidant activity of phycocyanin is related to the tetra chromophore and protein backbone^20^. On the other hand, chlorophyll a is associated to the structure, configuration, and polarity^21^. The antioxidant activity of coated instant rice was observed in terms of FRAP. It was observed that FRAP values increased significantly as the level of fortification with *S. platensis* increased (*p* < 0.05) by 1.6–6.8 mg Fe^2+^/g. Phycocyanin caused the reduction of the Fe^3+^/ferricyanide complex to ferrous form and the Fe^2+^ can be observed^20^. This result is consistent with that of Sengupta et al.^18^ who added *S. platensis* to soy yogurt. The antioxidant capacity of coated instant rice might be due to the presence of phycocyanin, chlorophyll a, TPC, tocopherol, and carotene, as well as minerals including selenium, zinc, and iron in *Spirulina*^18^.

### Change in texture profile analysis (TPA)

Hardness, adhesiveness, cohesiveness, springiness, gumminess, and chewiness were the texture attributes of instant rice determined by TPA (Table 4). Hardness, adhesiveness, springiness, gumminess, and chewiness of the decreased significantly after coating it with MD, CMC, and *S. platensis* (*p* < 0.05), while there was no significant difference in the cohesiveness value (*p* < 0.05) resulting from the addition of MD and CMC to the instant rice. The softening of the coated instant rice might have occurred after the addition of more water, which may come from the properties of hydrocolloids like MD and CMC. The water acted as a plasticizer, cleaving hydrogen bonds (–H) and forming new hydrogen bonds between the molecules of water and cross-linked chains of hydrocolloids^22^ and active compounds of *S. platensis*. The polysaccharides in *S. platensis* can be substituted by sulphate esters and methoxyl groups, and may also carry pyruvic acid that contributes to their hydrophilic and hydrophilic properties^23^. Polysaccharides can contribute to gelation due to their methoxyl content and the formation of intermolecular hydrogen bonds with the coating materials^23^. In addition, MD presents a high degree of conjugation when reacting, even though it has a higher molecular weight than CMC^15^.

### Changes in sensory acceptance

For sensory evaluation, instant rice (coated and uncoated) was rehydrated by microwave at 700 W for 3 min and then the evaluation was conducted according to the attributes shown in Table 4. The panellists gave the best score on the 9-point hedonic scale to uncoated instant rice and that coated with 2% (w/v) *S. platensis*. The admixture of *S. platensis* with coating materials changed the appearance and colour of instant rice from white to green, which resulted in the panellists awarding a lower score for the colour (*p* < 0.05). The addition of *Spirulina* has previously been noted as unsuitable for the colour of yogurt^13^ and pasta^14^. However, the panellists preferred the instant rice fortified with 2% (w/v) *S. platensis* in terms of appearance because the instant rice fortified with 3–5% (w/v) *S. platensis* had a dark green colour, while that fortified with 1% *S. platensis* had a light green colour with a speckle.

Some panellists detected an unfavourable odour in the instant rice fortified with 3–5% (w/v) *S. platensis*. An increase of *Spirulina* level results in the product having an unnatural flavour^14^. The score for the softness attribute of coated instant rice was higher than that for the control. This might be attributed to the physicochemical and texture properties of the *S. platensis* and hydrocolloids present in this coated instant rice. There were no significant differences in adhesiveness scores between uncoated and coated instant rice. The instant rice enriched with *S. platensis* (2% w/v) had a good sensorial score for overall acceptability (7.1 ± 1.1), slightly higher than that of the uncoated instant rice (6.6 ± 1.0).

## Conclusions

This study showed the beneficial effect of an edible coating containing *S. platensis* on the physicochemical, texture, and sensory properties of microwaveable instant rice. Coating technology was successfully applied to coat microwaveable instant rice with hydrocolloids fortified with *S. platensis* using a CCD. The optimal concentrations of the hydrocolloids were 1.0% MD and 20.0% CMC. The physicochemical properties of instant rice can be modified by coating it with MD, CMC, and *S. platensis*. Coating the instant rice with MD and CMC reduced the texture properties. Sensory evaluation showed that coating instant rice with MD, CMC, and *Spirulina* can generally enhance its flavour and softness to the satisfaction of consumers. In conclusion, edible coating technology would be a way to improve the acceptance of functional products containing edible algae.

## Materials and methods

### Samples and sample preparations

Polished jasmine rice was obtained from the Lanna Rice Research Centre (Chiang Mai University, Chiang Mai, Thailand) in 2018. The identification was done according to rice expert of Chai Mai Agricultural Research and Development Centre, Department of Agriculture (Chiang Mai, Thailand). The use of plants in the present study complies with international, national and/or institutional guidelines.

The rice samples were washed with tap water and immersed in water using a solid–liquid ratio of 1 : 2.0% (w/v) for 30 min, and then cooked in a cooker (Sharp, KS-ZT18, Thailand) for 30 min. The cooked rice was then soaked in cool water (4 °C) for 10 min in order to reduce stickiness. After that, the rice was dried in a hot air oven at 105 °C for 20 min and shifted to 60 °C until the moisture content reached 10% DW^24^. After the end of drying, the instant rice was removed from the dryer and placed in a thermal box for 1 h and packed in polyethylene packages which were stored at 4 °C until further use.

The *S. platensis* was kindly supported by Green Diamond Co., Ltd. (Chiang Mai, Thailand). Before the experiment, the fresh *Spirulina* was dried by microwave vacuum dryer (March cool, VR 100, Thailand) at 4800 W for 40 min^25^. The dried *S. platensis* was then ground on a knife mill, sieved (150 mesh), and stored in a desiccator at ambient temperature (30–33 °C).

### Production of instant rice coated with *S. platensis*

In this study, a CCD was applied to study the effect of the wall materials and their responses by using the minimum number of experiments and conditions. The wall material was prepared by weighing out CMC and MD to the mass presented in Table 1 into a beaker then adding 100 mL of distilled water. The mixture was stirred with a magnetic stirrer at 60 °C until the CMC and MD were completely dissolved. Subsequently, *Spirulina* powder (2% w/v) was weighed and added to the mixture for another 15 min. The coating material was then mixed with the instant rice at a mass ratio of 1 : 10 (w/w) and stirred magnetically in a coating pan (MS Scientific Instrument Co., Ltd., Chiang Mai, Thailand) for 30 min. The colour, coating efficiency, rehydration ratio, texture, TPC, antioxidant activity, phycocyanin content, chlorophyll a content, and sensory evaluation were investigated.

### Analysis

#### Proximate composition

The ash, fibre, moisture, and fat content of rice and *S. platensis* was assayed by Association of the Official Analytical Chemists methods^26^. The protein content was determined by the Kjeldahl method. The carbohydrate content was obtained by subtracting the sum of protein, ash, moisture, fibre, and fat, from 100.

### Determination of colour

A HunterLab L*, a*, b* system (MiniScan EZ, Virginia, USA) was used to directly read the colour of the coated instant rice.

### Coating efficiency (CE)

The CE of coated instant rice was determined by the method described by Dewettinck and Huyghebaert^27^ with slight modifications. Exactly 300 g of dry instant rice (W_1_) with 30 g of *S. platensis* and polymer solutions (W_2_) added was stirred in a coating pan for 30 min. Finally, coated instant rice was weighed (W_3_). The CE was calculated as follows (1):

1 $$CE \left(\%\right)= \left(\frac{{W}_{3}-{W}_{1}}{{W}_{2}}\right)\times 100.$$

### Release of TPC and antioxidant activity

#### Sample extraction

The coated instant rice (1 g) was extracted twice with 70% ethanol at a ratio of 1 : 10 (w/v). Each time, the mixture was kept on a mechanical shaker (Memmert, WNB 45, Schwabach, Germany) for 1 h at room temperature. Afterward, the extracts were combined and centrifuged at 4000 rpm for 20 min (Eppendorf 5430-R, Germany). The supernatants were then concentrated to dryness using a rotary evaporator (Büchi R-205, Flawil, Switzerland) at 35 °C. The dried extracts were redissolved in 5 mL of 50% ethanol and used as a crude extract for quantification of TPC, antioxidant activity, phycocyanin, and chlorophyll a.

### TPC release

The release of TPC was carried out according to Singleton et al.^28^. One-tenth of Folin–Ciocalteu phenol reagent in 1000 µL of water was added to 200 µL of test sample, then 800 µL of 2% CaCO_3_ was added; finally, the volume was adjusted to 10 mL with 60% methanol. After standing for 30 min, the amount of TPC was quantified by UV–Vis spectroscopy at 740 nm and expressed as mg of GAE/100 g of DW. The calibration curve was constructed by plotting colour formation at 740 nm against GA concentration (y = 0.1148x + 0.0011; *R*^2^ = 0.99).

### FRAP

The release of antioxidant activity in terms of FRAP was observed in the optical density (OD) at 593 nm^29^. The FRAP reagent (3 mL) (300 mM acetate buffer; pH 3.6, 10 mM TPTZ in 40 mM HCl, and 20 mM FeCl_3_, at a ratio of 10 : 1 : 1) was injected into 150 µL of sample and then left to stand for 30 min before OD measurement. The FRAP release was expressed in terms of mg of Fe^2+^/100 g of DW as comparing to a standard (y = 0.6647x + 0.0071; *R*^2^ = 0.99).

### Characterization of compounds

#### Phycocyanin content

The coated instant rice (40 mg) was mixed with 100 mM potassium phosphate buffer pH 7.0 (10 mL) prior to being kept at 4 °C for 12–14 h. The sample was then centrifuged at 3000 rpm for 5 min. The supernatant was collected and analysed for phycocyanin using high-performance liquid chromatography (HPLC). The phycocyanin of the coated instant rice was characterized by the following method mentioned by Bennett and Bogoarad (1973) through an HPLC system (Agilent Technologies, Santa Clara, CA, USA) equipped with a photodiode array detector (217 nm) and C_18_ reverse-phase column (Waters C_18_, 250 mm × 4.6 mm × 5.0 µm). The phycocyanin (20 µL) was separated using the gradient method and the following mobile phase composition: water (A) and acetonitrile (B) at a flow rate of 1.0 mL/min. The gradient programme started with 33% mobile phase B and was kept isocratic for 25 min, then rose to 55% B and held until 35 min, and finally changed to 90% B and kept for 65 min. At the end of the process, the column was re-equilibrated to the initial condition and stabilized for 5 min. The column temperature was set at 40 °C.

### Chlorophyll a content

The content of chlorophyll a was determined using the procedure given by Kumar et al.^30^ with slight modifications. Briefly, 5 g of sample was mixed with 20 mL of cold acetone and then transferred to an ultrasonic bath (37 kHz, 340 W, Elmasonic S40H, Elma, Singen, Germany) for 5 min. Supernatants were taken out and then centrifuged at 4500 rpm for 5 min. The supernatant was analysed for chlorophyll a content using HPLC. The filtrated samples (20 µL) were injected into an HPLC system equipped with a C_18_ reverse-phase column (Waters C_18_, 250 mm × 4.6 mm × 5.0 µm). The mobile phase consisted of 80% methanol (A) and ethyl acetate (B) as an eluent at a flow rate of 1.0 mL/min at 30 °C. Chlorophyll a was detected using a photodiode array detector (440 nm) in a gradient over 40 min. The gradient programme started with 80% mobile phase A for 2 min, then reduced to 50% and held until 25 min, and finally changed to 80% A and kept for 13 min.

### Texture analysis

The texture of the coated instant rice was measured with a texture analyser (TA-XT plus, Stable Micro Systems, UK) with a 100 mm stainless steel probe (5 mm diameter). One gram of reheated the coated instant rice was placed on a platform. The probe was 8 mm above the base plate. Pre-test, test, and post-test speeds were 1 mm/s, with a compression of 90% strain. Ten replicates of the sample were tested. The observed values including hardness, adhesiveness, cohesiveness, springiness, gumminess, and chewiness were reported as average values after eliminating the deviation curves.

### Sensory evaluation

Acceptance of the coated instant rice was evaluated by a group of 50 panellists (semi-trained) including students and staff of the faculty of Agro-industry, Chiang Mai University, in the range of 20–50 years old. The panellists were asked to rate samples on a sheet of 9-point hedonic rating tests (9 was like extremely and 1 was dislike extremely) accordingly for overall acceptability^31^. The panellists were asked to judge the quality in terms of appearance, colour, seaweed aroma, overall taste, seaweed flavour, rice flavour, softness, adhesiveness, and overall acceptability. Ethical approval for this study was granted by the Chiang Mai University Research Ethics Committee (CMUREC No. 63/104). The informed consent form was received and signed from all participants. The entire study involving human participants was also done according to the declaration of Helsinki.

### Statistical analysis

All experiments were carried out according to the relevant guidelines and regulations. The data are shown as the mean and standard deviation for triplicate analyses. Design-Expert version 6.0.10 (Stat-Ease Inc., Minneapolis, MN, USA) was applied to perform the experimental design and the data analysis. The assessment of parameters in the mathematical model (Eq. 2) was carried out at a 95% confidential level.

2 $$Y= {\beta }_{0}+\sum {\beta }_{i}{X}_{i}+\sum {\beta }_{ii}{X}_{i}^{2}+\sum {\beta }_{ij}{X}_{i}{X}_{j},$$

where *Y* represents the response variable, ${\beta }_{0}$ is the interception coefficient, ${\beta }_{i}$ is the coefficient for the linear effect, ${\beta }_{ii}$ is the coefficient for the quadratic effect, ${\beta }_{ij}$ is the *ij*th coefficient of the interaction effect, and ${X}_{i}{X}_{j}$ are input variables that influence the response variable *Y*. Statistical significance was analysed at *p* ≤ 0.05 using Duncan’s multiple range tests using ANOVA in SPSS version 17.0 (SPSS Inc., Chicago, USA). The graphical figures were created and modified in Microsoft PowerPoint (Microsoft Office 2013, Washington, USA).

### Ethics

The study involving “Development of instant rice coating with *Spirulina* (*Spirulina platensis*)” was reviewed and approved by Dr. Liwa Padthaisong (Chairperson of the Chiang Mai University Research Ethics Committee) of Chiang Mai University (Approval ID: CMUREC No. 63/104).

## Data Availability

The data generated during the current study are available from the corresponding author on reasonable request.
